# Identification and validation of genes involved in cervical tumourigenesis

**DOI:** 10.1186/1471-2407-11-80

**Published:** 2011-02-22

**Authors:** Thangarajan Rajkumar, Kesavan Sabitha, Neelakantan Vijayalakshmi, Sundersingh Shirley, Mayil Vahanan Bose, Gopisetty Gopal, Ganesharaja Selvaluxmy

**Affiliations:** 1 Dept. of Molecular Oncology, Cancer Institute (WIA), Chennai, India; 2 Dept. of Pathology, Cancer Institute (WIA), Chennai, India; 3 Dept. of Radiation Oncology, Cancer Institute (WIA), Chennai, India

## Abstract

**Background:**

Cervical cancer is the most common cancer among Indian women. This cancer has well defined pre-cancerous stages and evolves over 10-15 years or more. This study was undertaken to identify differentially expressed genes between normal, dysplastic and invasive cervical cancer.

**Materials and methods:**

A total of 28 invasive cervical cancers, 4 CIN3/CIS, 4 CIN1/CIN2 and 5 Normal cervix samples were studied. We have used microarray technique followed by validation of the significant genes by relative quantitation using Taqman Low Density Array Real Time PCR. Immunohistochemistry was used to study the protein expression of MMP3, UBE2C and p16 in normal, dysplasia and cancers of the cervix. The effect of a dominant negative UBE2C on the growth of the SiHa cells was assessed using a MTT assay.

**Results:**

Our study, for the first time, has identified 20 genes to be up-regulated and 14 down-regulated in cervical cancers and 5 up-regulated in CIN3. In addition, 26 genes identified by other studies, as to playing a role in cervical cancer, were also confirmed in our study. UBE2C, CCNB1, CCNB2, PLOD2, NUP210, MELK, CDC20 genes were overexpressed in tumours and in CIN3/CIS relative to both Normal and CIN1/CIN2, suggesting that they could have a role to play in the early phase of tumorigenesis. IL8, INDO, ISG15, ISG20, AGRN, DTXL, MMP1, MMP3, CCL18, TOP2A AND STAT1 were found to be upregulated in tumours. Using Immunohistochemistry, we showed over-expression of MMP3, UBE2C and p16 in cancers compared to normal cervical epithelium and varying grades of dysplasia. A dominant negative UBE2C was found to produce growth inhibition in SiHa cells, which over-expresses UBE2C 4 fold more than HEK293 cells.

**Conclusions:**

Several novel genes were found to be differentially expressed in cervical cancer. MMP3, UBE2C and p16 protein overexpression in cervical cancers was confirmed by immunohistochemistry. These will need to be validated further in a larger series of samples. UBE2C could be evaluated further to assess its potential as a therapeutic target in cervical cancer.

## Background

Cervical cancer is the second most common cancer among women worldwide and the most common cancer in Indian women [1]. In most developing countries there are no organized screening programmes, as a result most patients report to tertiary centres in locally advanced stages.

Human papilloma viruses (HPV) have been shown to play a major role in the pathogenesis of cervical cancer, but it alone is not sufficient [2]. Additional events, activation of proto-oncogenes and inactivation of tumour suppressor genes, are required in the induction of cervical cancer.

Cervical cancer goes through a series of pre-malignant stages - Cervical Intraepithelial Neoplasia (CIN) 1, 2 and 3. In general it takes upto about 10 - 15 years for the normal cervical epithelial cell to become a malignant one. However, some CIN2 lesions may develop soon after HPV infection, suggesting that there could be alternate pathways involved. CIN1 and 2 have a higher rate of spontaneous reversion compared to CIN3 [3]. The CIN3 then progresses to invasive carcinoma, which can then metastasize to regional lymph nodes and distant organs (e.g. lung).

The advent of microarray based technology has helped study the expression patterns of more than 40,000 genes at a time [4]. Several groups have used microarray based technology to look for differentially expressed genes in the different stages of cervical tumorigenesis [5,6]. Few studies have followed up and validated the microarray data in a large number of genes [7,8]. The objective of our study was to identify genes differentially expressed between normal cervix, CIN1/CIN2, CIN3/CIS and invasive cervical cancer, using oligo-microarray technique, validate the genes so identified using Relative quantitation Real Time Polymerase Chain Reaction (RQ-RT-PCR) and detect potential biomarkers for early diagnosis and therapeutic targets.

## Methods

Archival total RNA extracted from punch biopsy samples from patients with cervical cancer, collected in RNA later (Ambion, Austin, USA; Cat no: AM7021) and stored in the tumour bank after an informed consent were used, after obtaining the Institutional Ethical committee's approval for the study. The RNA had been extracted from the biopsy samples using the RNeasy RNA extraction kit (Qiagen, Gmbh, Hilden; Cat no: 74106) as per the manufacturer's instructions.

Twenty eight cervical cancer patients' samples were included in the study. The criteria for inclusion in the study were as follows: 1. good quality RNA as assessed by Bio-analyser (RIN 6 or above); 2. paired paraffin block having at least 70% tumour cells; 3. sufficient quantity of RNA be available; 4. patient should have completed prescribed radiotherapy and follow-up information till death/last disease free status be available.

In addition, 5 normal cervix tissues from women who underwent hysterectomy for non-malignant conditions or for non-cervical cancer were included. Four CIN1/CIN2 and 4 CIN3/CIS (one CIN3/CIS was included for RQ-RT-PCR analysis directly) were also included after informed consent. The Normal and CIN samples underwent frozen section to confirm their histopathologic status and the samples were immediately snap frozen in liquid nitrogen. RNA was extracted from the samples using the RNeasy RNA extraction kit, as described above.

### HPV Testing

The quality of the DNA was assessed by amplifying for β globin and only then HPV testing was done using GP5+ and GP6+ primers [9]. HPV16 and 18 typing was done using Nested Multiplex Polymerase Chain Reaction (NMPCR) technique [10]. SiHa DNA for HPV16 and HeLa DNA for HPV18 (positive controls) and C33A DNA (negative control) were included in all runs.

### Microarray experiment

1 μg of total RNA from the tumour/CIN/Normal sample and universal RNA (Stratagene; Cat no: 740000-41) were reverse transcribed using Arrayscript at 42°C for 2 hrs to obtain cDNA using the Amino Allyl MessageAmp II aRNA amplification kit (Ambion, Austin, USA; Cat no: AM1797). The cDNA was amplified by in-vitro transcription in the presence of T7 RNA polymerase; aRNA thus obtained was purified and quantitated in NanoDrop (NanoDrop Technologies, Wilmington, DE, USA). 20 μg of tumour/CIN/Normal aRNA was labelled using NHS ester of Cy5 dye and the control universal aRNA was labelled using NHS ester of Cy3 dye. The Cy3 and Cy5 labelled aRNA was used for hybridization onto the microarray chips from Stanford Functional Genomics Facility (SFGF, Stanford, CA) containing 44,544 spots, for 16 hrs in Lucidea SlidePro hybridization chamber (GE Health Care, Uppsala, Sweden) at 42°C. After hybridization, slides were washed in 0.1× SSC, 1× SSC followed by 0.1× SSC and dried.

The slides were scanned in ProScanArray (PerkinElmer, Shelton, CT, USA). Griding was done using Scan array Express software package (version -4). The integrated or mean intensity of signal within the spot was calculated. The files were saved as GPR files.

All the raw data files have been submitted to GEO with an assigned GEO accession number - **GSE14404**.

### Microarray dasta analysis

The Foreground Median intensity for Cy3 and Cy5, Background Median intensity for Cy3 and Cy5, spot size data were imported into BRB-ArrayTools software [11] using the Import wizard function. Background correction was not done. Global normalization was used to median centre the log-ratios on each array in order to adjust for differences in labelling intensities of the Cy3 and Cy5 dyes. The data was analysed using the Class comparison and Class prediction modules in the BRB-Array Tools software. In addition, Lowess normalization was also done separately and the data analysed using the modules mentioned above. The normalized Log ratios were also imported into Significance Analysis of Microarray (SAM) [12] software and analysed.

### Class Comparison in BRB-Array Tools

We identified genes that were differentially expressed among the four classes (Normal, CIN1/2, CIN3/CIS, Cancer) using a random-variance t-test. The random-variance t-test is an improvement over the standard separate t-test as it permits sharing information among genes about within-class variation without assuming that all genes have the same variance [13]. Genes were considered statistically significant if their p value was < 0.01. In addition a two fold difference was required between the Cancer and Normal, CIN3/CIS and Normal, CIN1/2 and Normal. The same was repeated with the Lowess normalized data using the same criteria.

### Class prediction in BRB-Array Tools

We developed models for utilizing gene expression profile to predict the class of future samples based on the Diagonal Linear Discriminant Analysis and Nearest Neighbour Classification [11]. The models incorporated genes that were differentially expressed among genes at the 0.01 significance level as assessed by the random variance t-test [13]. We estimated the prediction error of each model using leave-one-out cross-validation (LOOCV) as described [14]. Leave-one-out cross-validation method was used to compute mis-classification rate. From the list, genes were sorted further based on 2 fold difference between Cancer versus CIN1/2 & Normal, CIN3/CIS versus CIN1/2 & Normal, and CIN1/2 versus Normal. The same was repeated with the Lowess normalized data using a significance value of 0.01.

### SAM Analysis

The normalized log ratios of all the samples were imported into SAM software and analysed. A Multi-class analysis with 100 permutations was done. A delta value of 0.96 and a fold difference of 2 was used to identify the genes differentially expressed.

### Quantitative Real time PCR

High Capacity Reverse Transcription kit (Applied Biosystems, Foster City, CA; Cat no: 4368814) was used to reverse transcribe 2 μg of total RNA from the 38 samples in a 20 μl reaction volume. In 3 samples, due to the limiting amount of RNA, 0.75 μg was used for the cDNA synthesis.

These cDNA samples were used for real time PCR amplification assays using TaqMan^® ^arrays formerly TaqMan^® ^Low density arrays (TLDA) (Applied Biosystems, Foster City, CA; Cat no: 4342261). The fluorogenic, FAM labelled probes and the sequence specific primers for the list of genes with endogenous control 18S rRNA were obtained as inventoried assays and incorporated into the TaqMan^® ^array format. Quadruplicate (n = 38) and duplicate (n = 3; with limiting amount of RNA for cDNA synthesis) cDNA template samples were amplified and analysed on the ABI Prism 7900HT sequence detection system (Applied Biosystems, Foster City, CA).

The reaction set up, briefly, consisted of 1.44 μg of cDNA template made up to 400 μl with deionised water and equal amounts of TaqMan^® ^Universal PCR Master Mix (Applied Biosystems, Foster City, CA; Cat no: 4304437). 100 μl was loaded into each of the 8 ports of the array (2 ports comprise of one sample replicate on the array). Thus, the samples run as duplicates were only loaded into 4 ports of the array. Thermal cycling conditions included a 50°C step for 2 minutes, denaturation for 10 min at 94°C followed by 40 cycles consisting of 2 steps: 97°C for 30 seconds and 59.7°C for 1 minute for annealing and extension.

The raw data from the Prism 7900HT sequence detection system was imported into the Real-Time StatMiner™ software for statistical analysis of the data. Among the endogenous reference genes included on the array (18S ribosomal gene; UBC, β2 microglobulin), UBC and β2 microglobulin were chosen after visualizing the global Ct value distribution, for normalizing the data (Supplementary figure 1). The TLDA assays were run at LabIndia Instruments Pvt Ltd laboratories at Gurgaon, New Delhi.

#### Immunohistochemistry (IHC)

IHC was done for MMP3 protein expression in 5 Normal cervical tissue, 30 dysplasias of varying grade (CIN1 - 11; CIN2 - 8; CIN3/CIS - 11) and 27 invasive cervical cancers. A 3 layered ABC technique was used as described previously [15]. MMP3, monoclonal antibody (Sigma Aldrich, India; cat no: M6552) was used at a dilution of 1:75 and with wet antigen retrieval method. Positive control (section from a pancreatic cancer) and negative control (omission of primary antibody) were included in each run. The slides were scored by SS and TR independently and where discordant, jointly. The scoring was based on percentage of tumour cells immunoreactive (negative - 0; <25% = 1; 25-50% - 2; 51 - 75% - 3; >75% - 4), intensity of immunoreactivity (negative - 0; + - 1; ++ - 2; +++ - 3) and the compartment stained (cytoplasmic, nuclear or stromal). The scores obtained were added and the threshold was set at above the scores seen in the Normal cervical tissue (maximum score seen in Normal cervical tissue was 8). Hence tissues with a score of 9 or above were considered to overexpress MMP3.

p16 IHC was done as described previously [16] on 5 normal cervical tissue, 31 dysplasias of varying grades (CIN1 - 12; CIN2 - 8; CIN3/CIS - 11) and 29 tumours. Slides were scored as reported previously [16].

UBE2C IHC was done as above using wet autoclaving with a hold time of 5 minutes. Rabbit UBE2C polyclonal antibody (Millipore, USA - catalogue no: AB3861) was used at 1 in 100 dilution. The scoring was done similar to the scoring of MMP3 staining, with the maximum score seen in normal cervical tissue being 6. Hence a score of 7 or above was considered to be overexpression.

#### UBE2C in cervical cancer cell lines

Taqman Real time PCR was done for UBE2C levels in SiHa, C33A, HeLa, ME180, BU25K and HEK293 (Human embryonic kidney cells) cell lines. GAPDH was used to normalize the data.

Dominant negative UBE2C, in which Cysteine 114 is replaced by Serine, leading to loss of catalytic activity [17] was introduced into SiHa cells, using Fugene 6 Transfection Reagent (Roche Applied Science) according to the manufacturer's instructions using a 3:2 Fugene/DNA ratio. The effect on growth was assessed using the MTS assay (Promega) in the SiHa wild type (WT), in SiHa with pcDNA vector alone (SiHa pcDNA) and in SiHa with dominant negative UBE2C (SiHa DN-UBE2C).

#### Statistical analysis

Comparison between group means was assessed using a one-way ANOVA and multiple-comparison correction by Holm-Sidak method using Sigmaplot version 11.0. Fisher's exact test (2 tailed) was used to assess significance of IHC immuno-reactivity between cancer and dysplasias.

## Results

The stage distribution of the invasive cancer cases was as follows: IB - 2, IIA - 4, IIB - 18 and IIIB - 4. Twenty seven of the tumours were Squamous cell carcinomas (18 Large cell non-keratinizing, 5 large cell keratinizing and 4 unspecified) and one was a poorly differentiated carcinoma. Eighteen were HPV16 positive, 6 were HPV18 positive and 4 were HPV16 and 18 subtype negative (but HPV positive). All the Normals were HPV negative while one CIN1/2 and all the CIN3/CIS were HPV16 positive.

Using different methods, as described above, genes that were found to be differentially expressed between the four classes (Normal, CIN1/2, CIN3/CIS and Cancer) were identified. We did not use a Training set and a Test set for the Class Prediction model but used LOOCV for cross-validation and obtain the mis-classification error. The list of genes significant by different methods of microarray analysis is given in the Additional File 1 (AF1).

Sixty nine genes were selected for further validation by RQ-PCR using the Taqman Low Density Array card (TLDA) format (Additional File 2). These 69 genes formed part of the 95 genes selected for analysis using the TLDA format. The additional genes were those which had been found to be differentially expressed between the responders and non-responders to radiotherapy only treatment. Apart from the mandatory endogenous 18S rRNA included in the TLDA cards, based on the microarray data, UBC and β2 microglobulin, were included as additional endogenous reference genes.

Two of the samples CXL19-hov160 and CXM024-hov210 which had worked in microarray did not amplify satisfactorily in the RQ-TLDA assay and had to be removed from further analysis. In addition, RPS3A gene did not amplify in any of the samples.

The RQ values after calibrating with the Normal samples (Mean) for all the 94 genes showed 8 additional genes to be overexpressed; 4 (ASB16, CCL18, FST, THOC6) in Cancers, 1 (KLK9) in CIN3/CIS and 3 (RASSF6, TMEM123 and GLB1L3) in CIN1/2 samples. These 8 genes had initially been chosen for validation of the differentially expressed genes between responders and non-responders to radiotherapy. After excluding the genes which did not amplify, we now had 76 genes for further analysis.

Of the 31 genes which had been selected based on a greater than 2 fold difference between cancer versus CIN1/2 & Normal, 28 were concordant between the microarray data and the RQ-RT-PCR (Concordant rate of 90%). Three of four genes selected based on higher level of expression in Normals compared with all other classes showed concordance between the different methods of analysis. In the case of CIN1/2, concordance was seen in 6/7 genes (86%). However, with CIN3, this dropped to 41% (11/27). In four additional genes, there was a two fold greater difference between CIN3/CIS and Normal but not with CIN1/2. The overall concordance rate between the microarray data and the RQ-RT-PCR was 70% (48/69).

**Figure 1:**
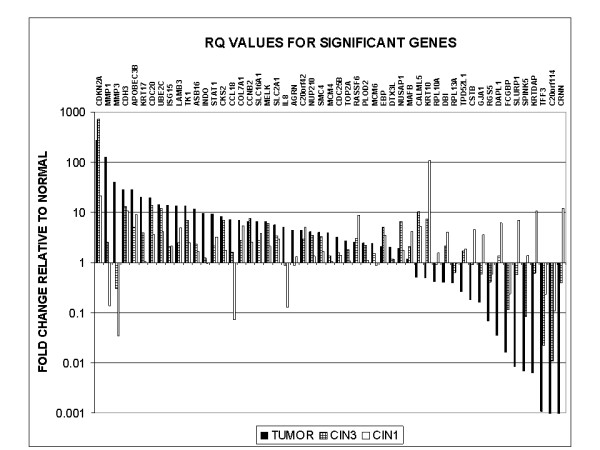
**Relative quantitation levels of significant genes**.

The list of genes validated and found to have a greater than 2 fold difference compared to the Normal, in the 3 different classes (Cancer, CIN3/CIS and CIN1/2) is given in Table 1. Figure 1 provides the fold change relative to Normal for these genes.

**Table 1:** Rq Values For The Genes Relative To Normal

	TUMOR	CIN3	CIN1	p VALUE	ADJUSTED p VALUE
**CANCER**					

AGRN	4.43991	0.87936	1.30918	1.95E-06	1.22E-05

APOBEC3B	28.7531	5.01379	8.85884	0.00203	0.00465097

ASB16	11.8884	2.29963	1.66354	0.01969	0.033056418

C20orf42/FERMT1	4.39903	2.94499	5.01419	3.16E-05	0.000123866

CCL18	7.20321	1.62004	0.0723	0.00717	0.013221849

CDC20	19.7377	13.8732	3.55849	2.98E-12	2.80E-10

CDC25B	3.2086	1.52287	1.4008	6.66E-05	0.000231975

CDH3	29.0774	13.1587	10.9346	4.79E-08	6.44E-07

CDKN2A	274.282	731.037	21.5102	2.75E-09	6.46E-08

CKS1B	2.13404	1.45612	0.69024	0.43779	0.587807354

CKS2	8.23126	6.96362	1.77975	2.75E-10	1.29E-08

COL7A1	7.1098	2.76264	5.36018	2.29E-06	1.34E-05

DTX3L	2.02309	1.16971	1.156	0.00341	0.006842128

FST	3.82365	0.71799	1.70232	0.04615	0.06996509

IGF2BP2	2.47182	0.45821	2.32119	0.13561	0.176463734

IL8	5.09435	0.86174	0.12926	0.00384	0.007519964

INDO	9.6009	1.25824	0.94788	0.00063	0.001856627

ISG15	13.9329	2.06597	2.16239	3.44E-07	3.23E-06

ISG20	2.11654	0.49943	0.3666	0.05737	0.080570517

KRT17	20.0558	3.92122	1.04329	1.42E-08	2.50E-07

LAMB3	13.6515	2.4397	4.96423	5.70E-06	2.82E-05

MCM4	3.85854	1.34001	1.07006	0.0004	0.001222519

MCM6	2.38446	1.48791	0.87143	0.00451	0.00865803

MELK	6.63844	5.96901	2.12471	1.75E-06	1.17E-05

MMP1	125.944	2.52253	0.1402	4.30E-07	3.67E-06

MMP3	40.0729	0.30275	0.03493	4.44E-05	0.000166947

NUP210	4.14316	3.45836	1.34658	0.00106	0.002782383

PLOD2	2.41806	2.21483	1.11276	0.00709	0.013221849

SLC16A1	6.71744	2.80011	3.82016	0.00092	0.002541873

SLC2A1	5.69119	3.34455	2.93698	0.00079	0.002242462

SMC4	3.93197	3.28125	1.65161	1.72E-05	7.68E-05

STAT1	9.1538	2.15413	3.16578	9.84E-05	0.000330443

THOC6	2.73991	1.63077	1.93361	0.08808	0.197141325

TK1	13.4525	7.11209	2.42871	4.91E-10	1.54E-08

TOP2A	2.72523	1.82496	1.08463	0.00164	0.003959245

UBE2C	14.054	12.1512	4.10121	3.51E-06	1.83E-05

C20orf114	0.00068	0.01085	0.1082	0.0002	0.000644363

FCGBP	0.01637	0.11346	0.23912	4.98E-05	0.00017995

***RGS5***	0.0692	0.40561	0.58543	9.63E-07	6.96E-06

***RPL10A***	0.42083	0.89019	1.54046	0.01077	0.019108208

***RPL13A***	0.39404	0.62725	0.96561	0.00199	0.00465097

***SPINK5***	0.00677	0.0817	1.38502	9.33E-06	4.39E-05

TFF3	0.00108	0.02275	0.23349	3.40E-07	3.23E-06

TPD52L1	0.26451	1.71067	1.8713	0.00218	0.004873472

**CIN3**					

CALML5	0.51194	10.2307	5.18399	0.00709	0.05123751

CCNB1	10.6825	10.44	2.79343	0.0175	0.090329125

CCNB2	6.74715	7.52496	2.54768	0.00064	0.008554526

EBP	2.05888	5.0445	3.49571	0.00027	0.008554526

FLJ44635	1.40964	6.98147	3.89307	0.22913	0.365061765

KLK9	11.7606	30.8271	26.324	0.01049	0.065711523

NUSAP1	1.92037	6.57218	1.75057	0.00533	0.049372841

PCNA	1.76308	2.64579	0.92838	0.01586	0.090329125

**CIN1**					

***B4GALT1***	1.29547	0.63469	2.21031	0.05915	0.166469075

***CAPNS2***	0.32306	1.98671	2.3676	0.01723	0.095298509

***CD36***	0.71359	10.6158	14.142	0.00889	0.052209988

CRNN	0.00053	0.40085	12.1177	0.00104	0.027439506

CSTB	0.17911	0.90884	4.5624	0.00263	0.029029697

CXCL14	0.09773	1.49396	11.7379	0.07384	0.182664861

DAPL1	0.03534	1.35468	6.09254	0.00067	0.027439506

***DBI***	0.40666	2.15767	3.98554	0.00469	0.031486868

***DYNLL1***	5.20441	4.20119	6.97185	0.07896	0.185551839

***FBLN1***	0.15796	0.34282	2.85624	0.05894	0.166469075

GJA1	0.16263	0.60199	3.58862	0.0017	0.029029697

GLB1L3	0.11823	0.47805	5.23721	0.04676	0.16283811

***HEBP2***	0.82928	1.50603	2.15449	0.03338	0.144693437

***HOPX***	0.04259	0.57251	2.37465	0.07856	0.185551839

***KRT10***	0.50079	7.42996	107.277	0.00309	0.029029697

***KRTDAP***	0.00637	0.61177	10.8535	0.00431	0.031486868

***MAFB***	1.18718	2.08998	4.15406	0.0029	0.029029697

RASSF6	2.55599	3.073	8.63768	0.00189	0.029029697

SLURP1	0.00845	0.56737	7.04202	0.00117	0.027439506

TMEM123	2.77691	1.00492	19.4085	0.03995	0.150195313

**GENES WITH LESS THAN TWO FOLD DIFFERENCE IN THE 3 CLASSES**					

PARP14	1.61309	0.75545	0.6995	0.04808	0.071743665

RPS8	0.75787	0.87528	0.97209	0.59992	0.65977976

RPL9	1.34116	0.44416	0.59991	0.41918	0.49253415

TIMM8B	0.82901	1.51431	1.92649	0.55825	0.628406378

Gene symbols in bold italics indicate those which were not concordant between microarray and RQ-RT-PCR analysis

**Figure 2:**
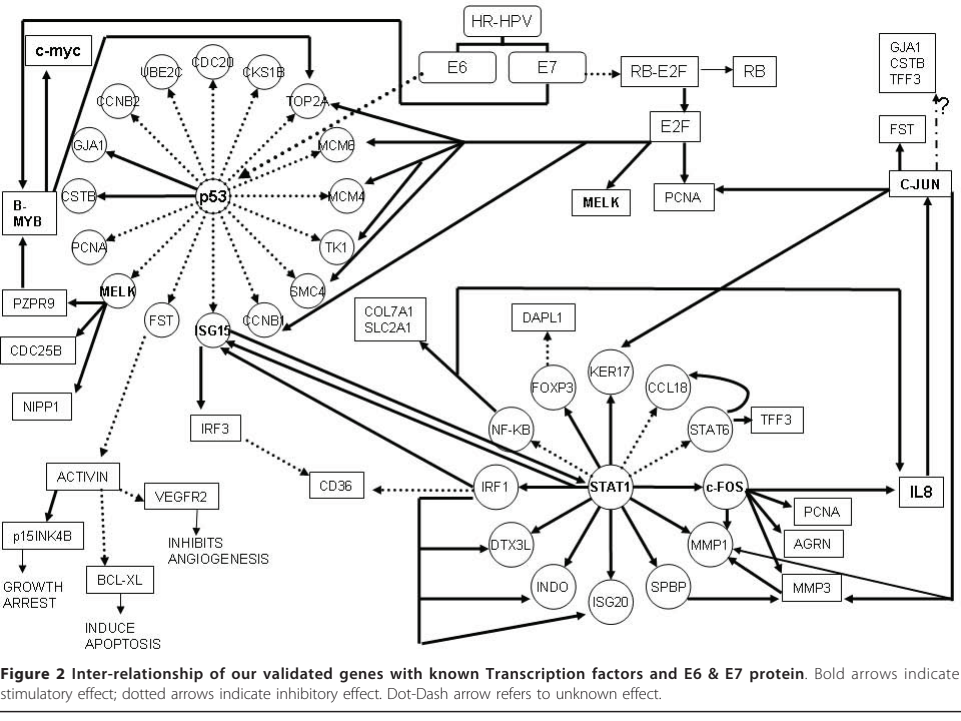
**Inter-relationship of our validated genes with known Transcription factors and E6 & E7 protein**. Bold arrows indicate stimulatory effect; dotted arrows indicate inhibitory effect. Dot-Dash arrow refers to unknown effect.

The genes were grouped on the basis of whether or not they were known to be involved in cervical tumorigenesis (Tables 2 and 3). Gene Ontology mapping was done using Babelomics software [18], which showed an over-representation of genes involved in cell cycle, cell division, catabolic process and multi-cellular organismal metabolic process. The genes identified to be differentially expressed were then analysed for specific pathways of relevance by manual curetting of data from published literature and online databases. The genes were grouped under the following categories: 1. Cell cycle regulatory genes (n = 13); 2. Interferon induced genes (n = 5); 3. Ubiquitin pathway (n = 5); 4. Myc Pathway [19] (n = 12); 5. HPV-E6/E7 related genes [20] (n = 14); 6. RNA targeting genes (n = 3) (details are given in Additional File 3). In addition, 40 genes in our list were found to be potentially regulated by p53 family of genes [21] (Additional File 4). Using GeneGo's Metacore software (Trial version) (url: http://www.genego.com), the relationship of our validated genes with known Transcription factors was analyzed. Based on this and from the manually curetted information, we then attempted to construct relationship chart (Figure 2) providing information on the gene interactions.

**Table 2:** Genes Identified as Up or Down-Regulated In Cervical Cancers For The First Time

**S. No**:	GENE SYMBOL	FUNCTION	CANCERS WHEREIN UP-REGULATION REPORTED
1	AGRN	Basement membrane component	Hepatocellular carcinoma (HCC)[27], Synovial sarcoma [28]

2	CCL18	Attracts lymphocytes towards dendritic cell	Gastric cancer [29]

3	CKS1B	Cell cycle	Breast cancer [30] etc.

4	COL7A1	Epithelial basement membrane organization	Oesophageal cancer [31]

5	DNYLL1/DLC1	Inhibits neuronal nitric oxide synthase	Breast cancer [32]

6	FERMT1	Cell adhesion; TGF-β signalling	Lung and colon cancer [33]

7	FST	Activin antagonist; inhibits synthesis and secretion of FSH	Wilm's tumour, Basal cell carcinoma [34]

8	IGF2BP2	Regulate translation of target mRNA	Testicular cancer [35], HCC [36]

9	IL8	Inflammatory cytokine	Several cancers; HR-HPV+VIN [37]

10	KLK9	Serine protease	Bladder cancer [38], breast cancer [39]

11	MELK	Leucine zipper kinase	Colo-rectal cancer, lung cancer, [40] brain cancer [41]

12	PLOD2	Lysyl hydroxylase	Glioblastoma [42]

13	RASSF6	Tumour suppressor	Low levels detected in HeLa cell line [43]

14	SLC16A1	Monocarboxylate transporter	Neuroblastoma [44]

15	SMC4	Chromosome condensation; DNA repair	Breast cancer [45]

16	APOBEC3B	RNA editing enzyme	None to date

17	ASB16	Ubiquitin pathway	None to date

18	NUP210	Nuclear pore complex	None to date

19	THOC6	Splicesome associated protein	None to date

20	TMEM123	Induces Pro-oncosis type cell death	None to date

	**CIN3**		

1	CCNB2	Cell cycle	Lung [46], pituitary tumours [47]

2	EBP	ER Protein	ALK+ Anaplastic large cell lymphoma [48]

3	NUSAP1	Microtubule associated protein	Melanoma [49]

4	CALML5	Calcium binding protein	Psoriasis [50]

5	FLJ44635	TPT1-like protein	None to date

			**CANCERS WHEREIN DOWN-REGULATION REPORTED**

1	C20ORF114	Innate immunity	Nasopharyngeal cancer [51]

2	CRNN	Tumour suppressor	Tongue cancer [52]

3	CSTB	Thiol proteinase inhibitor; anti-metastatic	Laryngeal cancer [53]

4	DBI	Intracellular carrier for Acyl-COA esters	Overexpressed in Brain tumours [54]

5	FCGBP	Maintenance of mucosal surface	Prostate [55]

6	HOPX	Tumour suppressor	Choriocarcinoma [56] and lung cancer [57]

7	SLURP1	Anti-tumour; anti-angiogenic	Hypopharyngeal cancer; Can induce apoptosis in Kaposi's sarcoma [58]

8	SPINK5	Serine protease inhibitor	Tongue cancer [59]

9	TFF3	Protect mucosa	Down-regulated in highly invasive colon cancers [60] and in thyroid cancers [61]

10	CAPNS2	Thiol protease	None to date

11	DAPL1	Epithelial differentiation	None to date

12	GLB1L3	Galactosidase beta1 like	None to date

13	HEBP2	Heme binding protein	None to date

14	KRTDAP	Keratinocyte differentiation	None to date

**Table 3:** Genes Known To Be Up or Down-Regulated In Cervical Cancers Found Also In Our Study

UP-REGULATED			
**S NO**:	**GENE SYMBOL**	**FUNCTION**	**REFERENCES**

1	CCNB1	Cell cycle	Overexpressed in cervical cancers [62]

2	CDC20	Cell cycle	HR-HPV E2 interaction [63]

3	CDC25B	Cell cycle	FOXM1 increases Cyclin B1, CDC25B, Cyclin D1 in cervical cancers [64]

4	CDH3	Cell adhesion	P-Cadherin predominant cadherin in high grade cervical dysplasia [65]

5	CDKN2A	Cell cycle	Overexpressed in high grade dysplasia and invasive cancers [66]

6	CKS2	Cell cycle	Overexpressed in cervical cancer [67]

7	DTX3L	Ubiquitin pathway	Overexpressed in cervical cancer [68]

8	INDO/IDO1	Immuno-suppression	Role in inducing immunosuppression in the tumour milleu [69]

9	ISG15/G1P2	Ubiquitin like protein	Over-expressed in invasive cancer [70]

10	ISG20	Exonuclease with higher affinity for RNA	Up-regulated by HPV E6 [71]

11	KRT17	Intermediate filament; marker for epithelial "stem cells"	Overexpressed in cervical cancer [72]

12	LAMB3	Basement membrane protein	HR-HPV-E6 inhibits miR-218 which regulates LAMB3 [73]

13	MCM4	Cell cycle	Widely expressed in cervical cancers [74]

14	MCM6	Cell cycle	Over-expressed in cancer [75]

15	MMP1	Breakdown of extracellular matrix	Overexpressed in cervical cancers [76]

16	MMP3	Breakdown of extracellular matrix	Metastatic lymphnodes in cervical cancer harbour MMP3 positive tumour cells [77]; Increased in stroma in cancers [78]

17	SLC2A1	Glucose transporter	Expressed in CIN and invasive cervical cancers [79]

18	STAT1	Transcription activator	Overexpressed in cervical cancers [80]

19	TK1	Thymidine kinase	Up-regulated in invasive cancers [81]

20	TOP2A	Topoisomerase	Overexpressed in cervical cancers [82]

21	UBE2C	Ubiquitin pathway	Overexpressed in cervical cancer [76]

	**CIN3**		

1	PCNA	Cell cycle	Major up-regulation of PCNA upon progression to CIN3 [83]

**DOWN-REGULATED**			

1	FBLN1	Cell adhesion; tumour suppressor	E6 binds to Fibulin1 and modulates its activity [84]

2	CD36	Cell adhesion	CD36 down-regulated in high grade dysplasias and cancer [85]

3	CXCL14	Immunoregulatory cytokine	Down-regulated in cervical and head and neck cancers [86]

4	GJA1	Gap junction	Down-regulated in CIN3 and invasive cancers [87]

5	KRT10	Intermediate filament	Down-regulated in invasive cancer [72]

**Figure 3:**
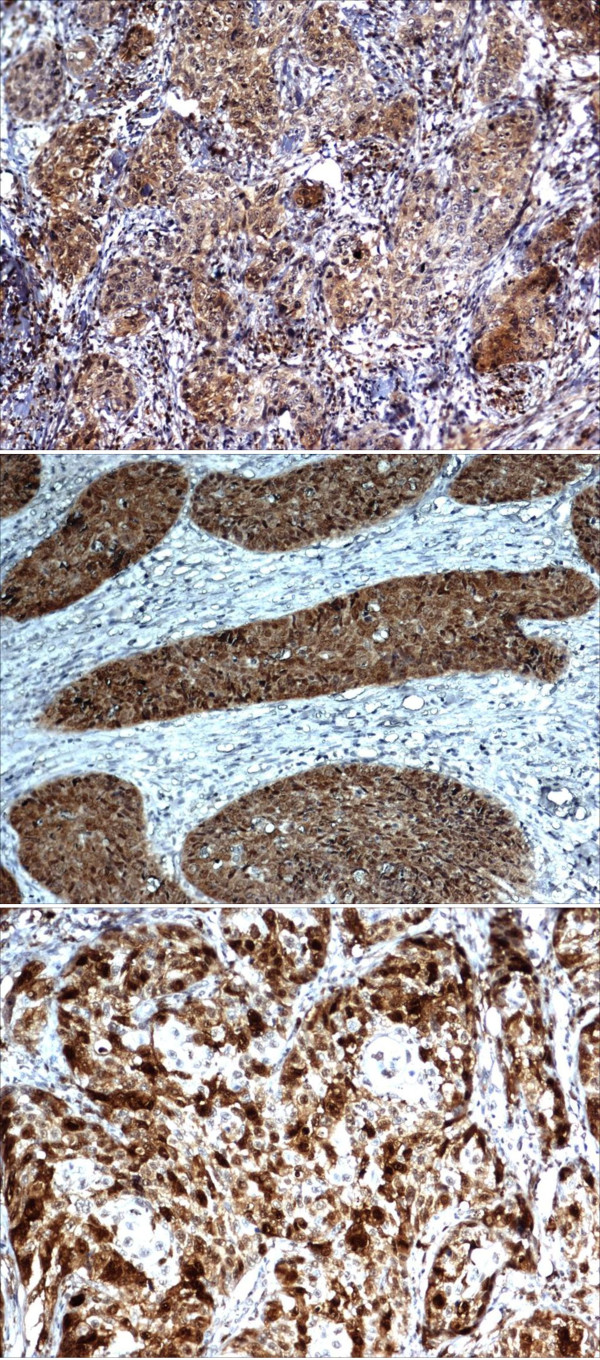
**Immunohistochemical staining for MMP3 (3A), p16 (3B) and UBE2C (3C) in invasive cancers (Magnification × 200)**.

Using IHC, we studied the protein expression for MMP3 in 5 normal cervical tissues, 30 dysplasias of varying grades and 27 invasive cancers. Using a semi-quantitative scoring system and a cut-off threshold set based on the normal cervical tissue staining, 6/30 dysplasias and 11/27 invasive cancers were found to overexpress MMP3 protein (Figure 3A). Among the patients whose tumours had been treated only with radical radiotherapy and had been followed up for a minimum period of 3 years, over-expression was seen in a greater number of tumours that failed treatment (6/9) compared to those free of disease at 3 years (2/12) (p = 0.03). p16 was found to be overexpressed in 19 of 31 dysplasias of varying grade and in 27/29 cancers (p = 0.005) (Figure 3B).

**Figure 4:**
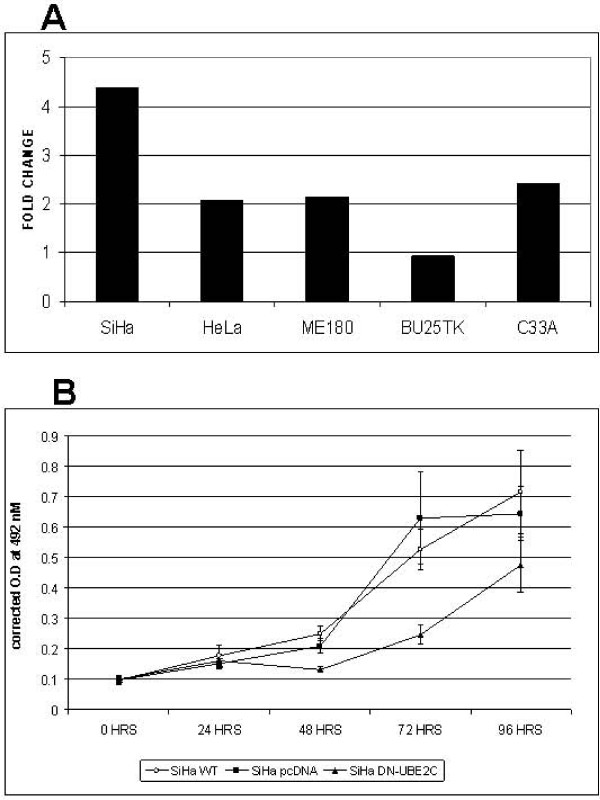
**UBE2C experiment data**. 4A: RQ of UBE2C in cervical cancer cell lines. Fold change relative to HEK293 cells. 4B: Growth curve for SiHa WT cells, SiHa cells transfected either with pcDNA alone or with Dominant negative UBE2C. ★ Denotes a statistically significant change (p < 0.001).

Using IHC, we found UBE2C to be overexpressed in 28/32 cancers, 2/11 CIN3/CIS and none of the CIN1 or 2 (Fisher's exact test p = 2.2 e^-11^) (Figure 3C). Using RQ RT-PCR, UBE2C was found to be overexpressed by more than 2 fold in SiHa, HeLa, C33A and ME180 relative to the HEK293 cells (Figure 4A). The growth of SiHa cells transfected with dominant negative UBE2C was significantly reduced at 48 and 72 hours compared to SiHa WT and SiHa transfected with pcDNA vector alone (p < 0.001) (Figure 4B).

## Discussion

There was good overall concordance between the microarray and the RQ-RT-PCR data. The lower concordance rate seen with the CIN3/CIS may be due to the additional CIN3 sample processed directly using RQ-RT-PCR. The relative quantitation values with and without the additional sample is given as Additional File 5. The concordance rate between microarray and semi-quantitative RT-PCR in the study by Gius et al [8] was less than 50%, using the standard microarray data analysis package.

There were several instances, wherein, a small difference in Microarray (above the 2 fold mandatory criteria) sometimes translated to large differences with RQ-RT-PCR (e.g. p16, MMP1, MMP3) and vice versa (e.g. CD36). This reinforces the point about the limitation of the microarray technique and it does emphasize the need for further validation, using assays like RQ-RT-PCR.

HPV16 was the predominant subtype seen in the invasive cancers and CIN3/CIS. However, we did not look for all the high risk subtypes and hence cannot exclude multiple subtype infection. Four of the cancers were HPV positive but HPV16 and 18 negative, suggesting that other high risk subtypes could be involved. None of the normal cervical tissues were HPV positive.

The genes that were for the first time, found to be over-expressed in cervical cancers compared to Normal cervix, is given along with information in which other cancers they have been reported to be overexpressed (Table 2A). Our study, for the first time, has identified 20 genes to be up-regulated in cervical cancers and 5 in CIN3; 14 genes were found to be down-regulated. In addition, 26 genes identified by other studies, as to playing a role in cervical cancer, were also confirmed in our study. UBE2C, CCNB1, CCNB2, PLOD2, NUP210, MELK, CDC20 were overexpressed in tumours and in CIN3/CIS relative to both Normal and CIN1/CIN2, suggesting that they could have an important role to play in the early phase of tumorigenesis. Among the genes which were up-regulated in cancers compared to that of Normal, CIN1/2 or CIN3/CIS, IL8, INDO, ISG15, ISG20, AGRN, DTXL, MMP1, MMP3, CCL18, TOP2A AND STAT1 are likely to play an important role in the progression of the disease.

STAT1 gene has a bi-phasic level, a rise in CIN1/2, drop in CIN3/CIS and a significant rise in invasive cancers. STAT1 has been considered generally to be a tumour suppressor, while STAT3 and STAT5 are known to be proto-oncogenes. However, recent studies have shown STAT3 to have both oncogenic and tumour suppressor function [22]. It could be that in cervical cancer, STAT1 may be protective in the early phase of HPV infection but could function as a proto-oncogene in the invasive stages of the disease. Highly invasive melanoma cell lines had high levels of STAT1 and c-myc [23].

The study by Lessnick et al., [24] showed that introduction of the potentially oncogenic EWS-FLI transcript into the fibroblasts, resulted in growth arrest rather than transformation. Knocking out p53 using HPV E6 helped overcome the growth arrest but was not sufficient to induce malignant transformation. The study used microarray to identify genes differentially expressed between the EWS-FLI transfected and the mock transfected cell line and found several genes related to growth promotion down-regulated. Our study had several genes [19] overlapping with theirs. Thirteen genes from our study were found to be HPV E6/E7 related genes[20] and 40 of the genes in our list were found to be potential p53 Family Target genes[21] (Additional File 3). In addition, there were 12 myc regulated genes, (MYC Cancer database at http://www.myc-cancer-gene.org/) of which CSTB which has been reported to be down-regulated by myc, was down-regulated in CIN3/CIS and in Cancer [19].

p16 gene, a tumour suppressor has been reported to be over-expressed in dysplasias and invasive cancer of the cervix. Several studies have tried to use this as a marker in the PAP smears for more reliable interpretation of the smear. von Knebel's group from Germany [25], had developed an ELISA to detect p16 in the cervical cell lysates, and reported a 96% sensitivity to pick up high grade dysplasias. Subsequently, the p16 ELISA assay was compared with Hybrid Capture 2 and was found to have comparable sensitivity and a slightly better specificity (46.9% versus 35.4%) [26]. Our RQ-RT-PCR data shows a gross over-expression of p16 in the CIN3 and invasive cancers (>250 fold). In our series of dysplasias and cancers, p16 protein was found to be overexpressed in invasive cancers compared to the dysplasias.

Figure 2 shows the inter-relationship of our genes with E6 and E7 protein and other known Transcription factors including p53, E2F, c-myc, B-MYB and c-Jun. The important genes in our list MELK, ISG15, STAT1, IL8, MMP1 and MMP3, could be playing critical roles in the tumorigenic pathway and could be potential targets for newer therapies.

UBE2C is an E2 enzyme involved in the process of ubiquitination. Townsley et al. [17] had developed a dominant negative UBE2C which lacks the catalytic activity. When the dominant negative UBE2C was expressed in SiHa cells, which have nearly 4 fold greater levels of UBE2C compared to HEK293 cells, it produced a significant growth inhibition (Figure 4B), indicating that the dominant negative UBE2C is competing with the wild type UBE2C, and can interfere with cell proliferation. Additional studies will be required to understand the mechanism by which this effect occurs.

## Conclusion

Our study has helped identify newer genes which could play a role in the cervical tumorigenesis and could offer the potential of developing newer diagnostic markers and therapeutic targets. We have confirmed over-expression of MMP3, UBE2C and p16 in tumours, by IHC. This will need to be validated further in a larger series of tumours and dysplasias. UBE2C will need to be studied further to assess its potential as a target for the treatment of cervical cancer.

## Conflict of interests

The authors declare that they have no competing interests.

## Authors' contributions

TR conceived the study; acquired, analysed & interpreted the data and drafted and revised the article. KS was involved in the acquisition and analysis of the microarray data. NV standardized and together with MB performed the microarray experiments and the immunohistochemistry. SS carried out all the pathological studies and assessment of samples for the microarray studies. GG standardized the UBE2C transfection into the SiHa cells and studied the effect on the growth of the cells. GS was involved in the clinical management and data analysis and follow-up of the patients. All the authors read and approved the final version of the manuscript.

## Pre-publication history

The pre-publication history for this paper can be accessed here:

http://www.biomedcentral.com/1471-2407/11/80/prepub

## Supplementary Material

Additional file 1 **List of genes differentially expressed identified by microarray analysis**. Click here for file

Additional file 2 **List of genes taken up for validation**. Click here for file

Additional file 3 **Identified genes linked to specific pathways**. Click here for file

Additional file 4 **p53 family regulated genes**. Click here for file

Additional file 5 **Relative Quantitation with and without CXM180**. Click here for file
